# Peptide-Based Hydrogels: Template Materials for Tissue Engineering

**DOI:** 10.3390/jfb14040233

**Published:** 2023-04-19

**Authors:** Roya Binaymotlagh, Laura Chronopoulou, Cleofe Palocci

**Affiliations:** 1 Department of Chemistry, Sapienza University of Rome, P.le A. Moro 5, 00185 Rome, Italy; 2 Research Center for Applied Sciences to the Safeguard of Environment and Cultural Heritage (CIABC), Sapienza University of Rome, P.le A. Moro 5, 00185 Rome, Italy

**Keywords:** peptide hydrogels, self-assembly, 3D scaffolds, tissue engineering

## Abstract

Tissue and organ regeneration are challenging issues, yet they represent the frontier of current research in the biomedical field. Currently, a major problem is the lack of ideal scaffold materials’ definition. As well known, peptide hydrogels have attracted increasing attention in recent years thanks to significant properties such as biocompatibility, biodegradability, good mechanical stability, and tissue-like elasticity. Such properties make them excellent candidates for 3D scaffold materials. In this review, the first aim is to describe the main features of a peptide hydrogel in order to be considered as a 3D scaffold, focusing in particular on mechanical properties, as well as on biodegradability and bioactivity. Then, some recent applications of peptide hydrogels in tissue engineering, including soft and hard tissues, will be discussed to analyze the most relevant research trends in this field.

## 1. Introduction

The final goal of tissue engineering is repairing and replacing injured tissues by providing human-made biocompatible constructs containing cells, biomaterials and bioactive molecules that are able to recreate the desired structures [1,2,3,4,5,6,7]. In recent years tissue engineering applications have been promoted significantly by the paramount advances in the fields of 3D/4D bioprinting [8,9,10], stem cells [11], novel genetic tools, [12,13] and biomaterials [14] and bioelectricity [15]. Biomaterials could provide 3D scaffolds, which should closely match the features of the physiological extra cellular matrix (ECM), and interact with desired cells to modulate cellular behaviors, giving rise to the generation of new tissue and organs [16,17,18]. Biomaterial scaffolds with an accurate design are crucial for tissue engineering development and applications.

**Figure 1:**
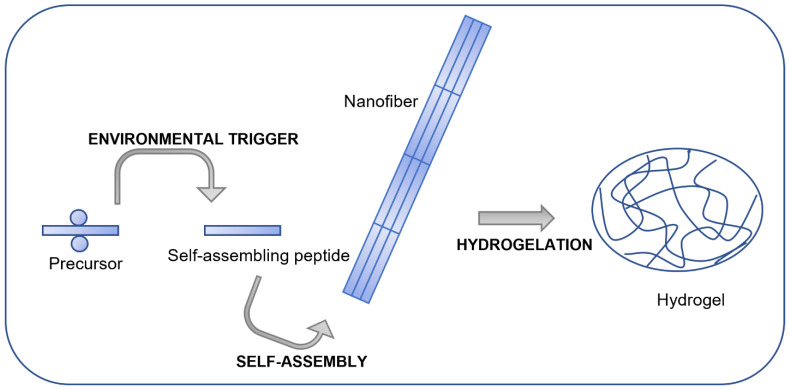
Scheme of the main steps involved in peptide hydrogel formation through self-assembly.

Hydrogel materials, consisting of 3D cross-linked macromolecular networks capable of entrapping a high amount of water molecules, could be good candidates for biomaterials for applications in tissue engineering [19]. In fact, hydrogels’ structure and composition are highly similar to the ECM, which enables entrapped cells to survive and proliferate [20,21]. Self-assembling peptides are an interesting class of hydrogels which could promote application in tissue engineering [22]. Peptides with appropriate sequences are able to self-assemble spontaneously, leading to the formation of porous hydrogels in definite environmental conditions (e.g., pH, ionic strength, temperature) (see Figure 1). The formed porous hydrogels can assume a wide variety of different morphologies such as nanotubes, nanovesicles and nanospheres, which can encapsulate cells and biochemical factors [23,24,25]. The molecular interactions present in these hydrogel scaffolds are usually non-covalent, such as van der Waals, electrostatic interactions, hydrogen bonds and π-π stacking. Various secondary structures (α-helix, β-sheet, β-hairpin) can be formed [26,27,28]. Such non-covalent structures with reversible assembly can lead to the formation of hydrogels which are self-healing [29,30], shear thinning [31,32] or with shape memory [33,34]. Due to the mentioned features, peptide hydrogels have significant potential to acquire a prevalent place as scaffolds in tissue engineering.

In this review, we will focus on some relevant properties for peptide hydrogels to be considered as 3D scaffolds and then discuss recent advances in their application for the regeneration of various tissues.

## 2. Main Features of Peptide Hydrogels as 3D Scaffolds

Peptide hydrogels need to possess some specific features to be applicable in tissue engineering as 3D scaffolds. For example, they need to possess adequate mechanical properties, as well as porosity and permeability, needed for cell seeding and growth. As it is clear, since these materials serve as temporary scaffolds in tissue engineering, biodegradability is also another important feature of hydrogels, and they should not hinder new tissue generation. Last but not least, bioactivity is essential for various regeneration applications [35,36]. As an example, for cardiac tissue repair, the electromechanical coupling of cardiac cells is crucial for ensuring the regular functionality of the heart tissue. Therefore, artificial scaffolds for cardiac tissue regeneration should have an acceptable conductivity [37]. As a result, the composition and structure optimization of peptide hydrogels is crucial to meet the requisites for tissue engineering.

### 2.1. Mechanical Features

Hydrogels are polymeric networks which have the ability to absorb a large volume of water; flexibility, versatility, stimuli-responsivity, and a soft structure are their main properties [3]. In particular, the viscoelastic behaviour of hydrogels is the property of the materials that exhibits both viscous and elastic characteristics when undergoing deformation. It is generated as a result of the conformational changes inside the polymeric structure which take place in order to find an equilibrium state when it is subjected to a stress. On this basis, some attention has been recently directed to tuning hydrogels’ viscoelasticity thanks to studies demonstrating that viscoelastic biomaterials regulate various aspects of cell behavior [20]. Viscoelastic biomaterials, notably hydrogels, provide time-dependent mechanical cues (i.e., stress relaxation) that affect cell behavior, including cell spreading, migration, proliferation, differentiation, and ECM deposition.

The mechanical characteristics of hydrogels are considered as key properties for assessing the possibility of the prepared constructs to be used as scaffolds for the regulation of cellular behaviors, which is based on mechanotransduction signal mediation [22,38,39,40]. Therefore, for withstanding physical stress in the physiological environment, identical mechanical characteristics of the scaffolds with healthy tissue or ECM are required [31,41,42,43]. In particular, the stiffness of hydrogels’ biomaterials is critical for the adjustment of cell behaviors [32,44]. However, sometimes preparing peptide hydrogels with enough stiffness to match the desired hard tissues is challenging. This may be related to some of their features such as low molecular weight, non-covalent interactions, and non-uniform and reversible self-assembly [45,46,47]. Nonetheless, it is possible to overcome these issues by modifying or functionalizing peptide hydrogels, by changing the parameters required for self-assembly, (i.e., peptide sequence, concentration, pH, temperature, ionic strength) [48,49,50,51,52,53,54,55,56,57]. Chemical crosslinking is an effective strategy for enhancing the mechanical properties of hydrogels, but it requires additional synthetic steps that may not be suitable for in situ hydrogel formation [58]. Functionalization with selected molecules is an alternative approach of improving mechanical properties, since appropriately chosen macromolecules could provide strong covalent interactions or co-assemble with peptides [59,60,61]. Some macromolecules, such as FDA-approved polyethylene glycol (PEG) [61,62,63,64,65,66,67], heparin [68], hyaluronic acid [69], alginate [70] and also proteins [71], may be incorporated in hydrogels, tuning their mechanical properties but also introducing new functions. In addition to stiffness, the viscoelasticity of the hydrogel is another important parameter [72]. For tuning hydrogel viscoelasticity, different approaches, such as the altering cross linker concentration [73] or the structure of the monomers [73,74], have been attempted. As is well-known, hydrogel elasticity refers to its capability to deform instantly, responding to a mechanical loading, and then to restore upon removal of the load. This property depends on the hydrogel’s intrinsic swelling properties which cause a penetration of a solvent into the polymer network, changing its volume. Such approaches are generally very useful to be applied for controlled drug release from hydrogel-based materials.

### 2.2. Biodegradability

**Figure 2:**
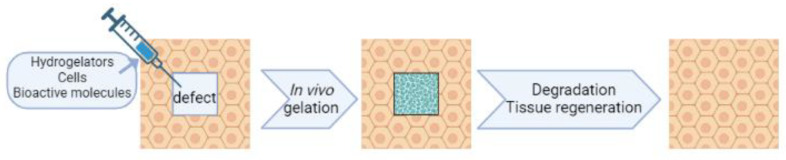
Schematic illustration of a biodegradable hydrogel for tissue engineering.

Overall, the presence of peptide hydrogels that are used as 3D scaffolds in tissue engineering and regeneration applications must be temporary. The degradation of peptide scaffolds simultaneously with the rate of tissue regeneration is desired [75,76] (see Figure 2). The ratio of the scaffold/tissue mechanical response is affected by scaffold degradation. Before its degradation, the scaffold is subjected to a mechanical load, which it must be able to bear without deformation occurring. When tissue growth initiates and progresses, the scaffold should start degrading at an appropriate rate, until the newly formed tissue ends bearing the mechanical stress on its own [77]. So, the degradation rate of the scaffold must be adjusted with that of tissue regeneration. In literature, different ways to provide biodegradable peptide hydrogels have been reported. One of them is using proteases such as endothelial cells-derived matrix metalloproteinase (MMP) [76,78,79,80,81,82]. Apart from cleavable sequences, the secondary structure of peptide hydrogels also affects MMP-based degradation. Since MMP collagenases only attack collagens [83,84,85,86] by changing the peptide sequences, the resulting hydrogels will become sensitive to other proteases, such as proteinase K (with broad cleavage activity), trypsin (that mainly hydrolyzes peptides at the carboxyl side of K or R aminoacids) [87,88], polymorphonuclear elastase (that usually cleaves at the carboxyl side of A, G and V aminoacids) [89] and papain (that preferentially cuts peptides after a K or R aminoacid preceded by a hydrophobic one and not followed by a V) [90]. With this strategy, the biodegradability of peptide hydrogels can be modulated for tissue engineering applications. However, we must mention that for designing peptide hydrogels with controlled degradation, the effect of other important parameters such as functional motifs and water uptake also needs to be taken into account [76,91].

### 2.3. Bioactivity

Another significant factor that is considered crucial for making peptide hydrogels applicable for tissue engineering is bioactivity. Bioactive peptide scaffolds provide suitable conditions for cell interaction, growth, migration, and differentiation. Peptide motifs derived from the ECM are capable of binding with integrins which can be found on the cell membrane and activate signaling pathways leading to ECM generation. The presence of bioactive peptides in the sequence of synthetic peptides could guide cells to assume desired cellular behaviors in appropriate environments [92,93]. In particular, peptides with small side chains are favored for ECM-mimicking because small motifs generally do not interfere with peptide self-assembly [94]. Various peptide motifs are valuable when fabricating bioactive peptide hydrogel scaffolds (see Table 1). In addition to peptide motifs, growth factors (GFs) also have an effect on the bioactivity of scaffold hydrogels. Attachment of GFs to peptide hydrogels leads to particular cell responses, which could increase the bioactivity of the scaffolds [95,96]. Despite their appreciated functions for covalent attachment to different peptide hydrogels [97,98,99,100,101,102,103,104], GFs are instable and their high costs limit their current clinical applications.

## 3. Peptide Hydrogel Scaffolds in Tissue Engineering Applications

Self-assembling peptide hydrogels are increasingly studied for tissue engineering applications thanks to their unique properties [125,126]. The nanofibrous microarchitecture of these hydrogels is more able to resemble the native ECM, compared to conventional hydrogels made from synthetic polymers [127]. So, they can be used as nanofibrillar scaffolds which create a biocompatible 3D microenvironment for host cells. Moreover, structural changes of these scaffolds can be performed by incorporating various functional peptide sequences, a relatively simple approach to enhance the biological effectiveness of this type of hydrogels. It has been investigated that self-assembling peptide hydrogels with β-sheet structure play a critical role in tissue engineering. Hydrophobic/hydrophilic and electrostatic interactions between amino acids motifs are the most common features of those self-assembling peptides with a β-sheet structure (Table 2) [1]. In the next paragraphs, we will review the recent development of self-assembling peptide hydrogels as 3D scaffolds in tissue engineering applications [128].

### 3.1. Angiogenesis and Vascularization

**Figure 3:**
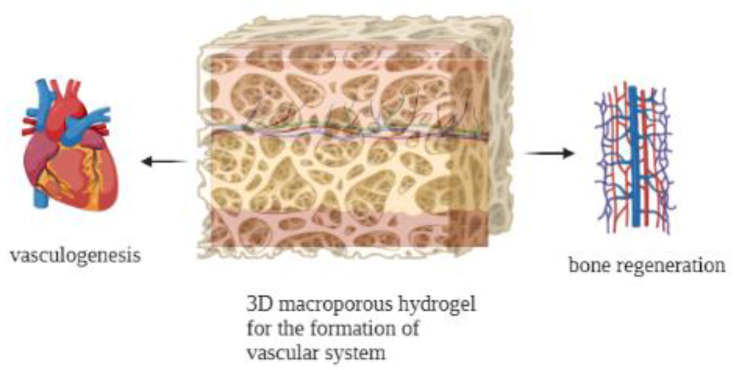
Scheme depicting the role of hydrogels in vascularization. Vascularized tissues inside hydrogels enable them for tissue regeneration.

As a fundamental process in tissue engineering and regeneration, angiogenesis relates to the sprouting of new blood vessels from pre-existent ones, including a series of highly dynamic and complex interactions among the supporting cells and growth factors [135,136]. Angiogenesis is a key process during tissue repair following ischemic diseases, because for every regrowth of damaged cells and tissues there is a necessity to establish an adequate blood supply [137]. Besides, vascular networks are vital for carrying bioactives, cell nutrients and oxygen to regenerate the damaged tissue [138]. Recently the significant advancement in hydrogels fabrication along with deep studies in vascular biology paved the way for designing three-dimensional (3D) tissue and organs comprising a highly complex vascular system [139] (see Figure 3). In 2021 Roy et al. reported the incorporation of an RGD peptide motif and an antivascular endothelial growth factor receptor-2 (VEGF-R2) DNA aptamer into a thiolated hyaluronic acid (HA) polyethylene diacrylate hydrogel to prepare a bifunctional scaffold [140]. Their results showed that RGD peptides improved cell growth whilst the DNA aptamer promoted cell viability, triggered cell migration and initialized angiogenesis. This study shows that these scaffolds can be studied for wound healing applications. Zhang et al. prepared RATEA16 hydrogels and reported the viability of human umbilical vein endothelial cells (HUVECs) and human stem cells of the apical papilla (SCAPs) seeded in this scaffold [141]. The authors also developed RATEA16-based drug delivery systems and studied the release features of VEGF and BMP-2 from the scaffold. The in vitro effect of the scaffolds on HUVECs angiogenesis was assessed, confirming the capacity of this scaffold to ensure HUVECs and SCAPs survival and promote angiogenesis. In addition, the drug-loaded scaffolds demonstrated biodegradability and biocompatibility. In another study, RADA16 was modified with QLK and LRK [142]. In this system, QLK sequence was used to begin crosslinking by endogenous transglutaminase and improve mechanical properties, while LRK motif was employed to bind heparan sulfate (HS) [24]. HS is a main component of glycosaminoglycans present in the ECM, and it is able to preserve the activity of GFs by preventing their enzymatic degradation [128,129]. The authors showed that the HS-modified scaffold provided a controlled and prolonged release of two GFs for 28 days. The HUVECs-seeded scaffold with the entrapped GF formed a tube-like structure *in vitro*, showing a fast hemostasis within 10 s in a rat defibrinated blood model. Moreover, the proangiogenic features of this material were studied. Results confirmed the blood vessel growth in chicken chorioallantic membrane [3].

Cell-free peptide scaffolds with angiogenetic properties were also designed and studied. For instance, Dos Santos et al. reported a cell-free and growth factor-free hydrogel containing elastin-like polypeptides (ELPs), PEG and IKVAV peptide [143]. Zhou et al. reported the use of an MMP-2 self-assembling peptide for the delivery of MSC-derived extracellular vesicles [144]. The in vitro response of MMP2, allowed for the extracellular vesicles release, increasing endothelial cell proliferation, and promoting angiogenesis within the hydrogel implanted in an injured tissue. PRG (PRGDSGYRGDS) and KLT (KLTWQELYQLKYKGI) are two different functional motifs, used for enhancing, respectively, cell adhesion and vascularization. The angiogenetic ability of RADA16/PRG and RADA16/KLT self-assembling peptides both in vitro and in vivo in a mouse model was evaluated [145]. RAD/KLT and RADA16/PRG peptide mixtures with bone marrow MSCs (BMSCs) were also studied in a mouse model for the treatment of acute myocardial infarction [146]. The presence of RADA16/PRG improved the localization and survival of BMSCs in the infarcted myocardium. KLT, a peptide mimicking VEGF, was studied to enhance the biological activity of peptide amphiphile (PA) molecules [147]. The authors showed, in in vitro studies, that VEGF-PA improved proangiogenic activity in endothelial cells through the selective activation of VEGF receptors. For the in vivo study of this system, the authors used nanofiber VEGF-PA gels in a mouse ischemia model, showing an increase in microcirculation density and functional recovery [128]. In 2008, Wang et al. modified RADA16 by functionalizing it with the KLT or the PRG motif [148]. These two nanofiber scaffolds showed proangiogenic potential and significantly improved endothelial cell proliferation, tubulogenesis and migration *in vitro*.

Overall, none of the synthetic peptide scaffolds described in the literature possesses angiogenic properties alone. Therefore, peptide functionalization with appropriate bioactive sequences or the incorporation of GFs are the two main successful strategies for inducing vascularization. Cell-free scaffolds are also very promising systems that may overcome some difficulties faced when transplanting exogenous cells [149]. In addition to the composition and structure of scaffolds, the bioavailability, biodegradability, and route of administration must be detected related to distinct tissue type.

### 3.2. Neural Tissue Engineering

The nervous system is considered as one of the most complex organizations of the body, and it consists of two parts: the peripheral nervous system (PNS) and the central nervous system (CNS). The damage of the nervous system threatens human health and can result in permanent and serious neurological deficiencies and even death, due to its limited regeneration potential. Thus, repairing injured neural tissues is a major challenge for scientists [150]. The advancement of cellular therapies has provided promising therapeutic strategies for repairing the nervous system, in which biomimetic self-assembling peptide hydrogels may act as a favorable microenvironment to improve the activity of transplanted cells as well as promoting the healing of damaged tissues [151,152]. In recent years, the interaction of self-assembling peptide hydrogels with neurons has been investigated in vitro [128]. To facilitate the repair of the CNS several factors should be considered, including enhancing angiogenesis, lessening the generation of glial scar tissue as well as concurrent inflammation processes. Although there is a plethora of studies on self-assembly systems for CNS regeneration, both in vivo and in vitro, no complete functional recovery has been observed to date.

#### 3.2.1. Peptide Hydrogels

Chai et al. [153] synthesized a temperature-sensitive peptide hydrogel decorated with IKVAV with a regular 3D porous structure, good biological activity, and rapid (de)swelling performance. The authors used this scaffold to treat spinal cord injury and showed its improved angiogenesis, inhibition of keratinocytes differentiation and adhesion, reduction of glial scar tissue generation. Their work demonstrated that this hydrogel is high-performing, promoting angiogenesis, and reducing the production of pro-inflammatory cytokines. More importantly, the biomaterial prevented the generation of glial scar tissue, which resulted in the healing of the damaged tissue. Wiseman et al. [154] synthesized a unique self-assembling peptide hydrogel, Fmoc-DIKVAV, as a valuable candidate for cell and drug delivery systems to brain tissues. The Fmoc group, containing an aromatic moiety, was used as it was known to promote supramolecular aggregation, thanks to the establishment of π-π interactions among aromatic groups. The authors studied the application of this scaffold in Fischer F344 rats for delivering mesenchymal precursor cells after mild thoracic contusion spinal cord injury. They showed that the Fmoc-DIKVAV scaffold could provide a beneficial microenvironment to promote cell infiltration and axonal regrowth. Hivare et al. synthesized an IKVAV-grafted DNA hydrogel using a chemical crosslinker [155]. They reported that the functionalized hydrogel scaffold was associated with a prolonged neurite length, enhanced neuronal differentiation, dynamic movement of cytoskeleton and microtubules, and changed endocytosis processes in the associated stem cells. Zhang et al. [156] synthesized a peptide hydrogel suitable for preparing an artificial neurovascular microenvironment by grafting the brain-derived neurotrophic factor (BDNF) and the vascular endothelial growth factor (VEGF). The hydrogel improved the neurite offshoot of pheochromocytoma cells (PC12) and the formation of tubular arrangements of HUVECs *in vitro*. Moreover, the in vivo tests in a rat brain lesion model evidenced a promoted fast cell infiltration in the injured tissue. The authors showed that this hydrogel initiated an effective mutual regulation of the production of paracrine factors from neural and vascular cells in indirect co-culturing experiments. Furthermore, for the direct co-culturing of the two cell types, an enhanced communication among the two cell types was detected that promoted the differentiation and maturation of both PC12s and HUVECs. Thus, this dual-functionalized hydrogel was successfully tested for the formation of a synthetic neurovascular microenvironment for regulating the properties of neural and vascular cells, improving their mutual interactions and communication by direct cell—cell contact and paracrine signaling. Wang and co-workers [120] reported the synthesis of a peptide hydrogel modified through the chemical attachment of a short functional motif with the C-terminus of RADA16. In particular, the SNVI motif (SNVILKKYRN), having BMP-7 bioactivity, was used for the preparation of the novel RADA16-SNVI peptide. The authors used this hydrogel for culturing adipose-derived stem cells (ADSCs): the hydrogel showed a good biocompatibility and triggered cell differentiation. Compared with control cells, ADSCs grown in the RADA16-SNVI scaffold showed a higher formation of the ECM marker collagen type II and aggrecan. For these cells (in SNVI-RADA16 gel), the balance between aggrecan and collagen was found to be about 29:1 after 21 days. Additionally, the results demonstrated that the gel supported the differentiation of ADSCs into nucleus pulposus-like cells, qualifying this system as an optimal material for neural tissue engineering applications. Another study developed a peptide-based hydrogel which is able to mimic the hydrophobic surface of a jigsaw-shaped moiety of glycophorin A, as a synthetic ECM for brain regeneration [157]. The authors showed that the peptide could form several micrometer-long supramolecular nanofibers that gave rise to a hydrogel in physiological conditions, which allowed the efficient incorporation of VEGF and its sustained release. Moreover, in cell-free experiments restorative effects were observed in a mouse stroke model. Other researchers synthesized an RGD/IKVAV-grafted RADA16 peptide [158] to promote the differentiation of neural progenitor cells/stem cells into neurons and astrocytes, and improve axons regeneration in a sciatic nerve defect model. The authors demonstrated that these amphiphilic peptide nanofibers are suitable scaffolds for regenerative applications [159,160]. Recently, a study reported the modification of the RADA16 peptide with the functional motif SVVYGLR, possessing the ability of promoting cell adhesion, migration, and differentiation [161]. In a zebrafish brain injury model, this hydrogel showed its capability to improve both neurogenesis, angiogenesis, and tissue regeneration. In recent years, artificial nerve guidance conduits have been studied for curing injuries with an extended gap [162]. For instance Zhan et al. used RADA16 as an intraluminal filler and implanted it to repair a 10 mm nerve gap after a sciatic nerve transverse cut [163]. This work showed that the RADA16 scaffold enhanced axonal remyelination and regeneration, as well as functional regaining.

#### 3.2.2. Hydrogels Made of Peptides and Organic/Inorganic Components

The functionalization of PAs with IKVAV and RGD sequences (IKVAV-PA, RGD-PA) aligned with poly(lactic-co-glycolic acid) (PLGA) for promoting schwann cells proliferation was reported [160]. When a PLGA/RGD-PA hydrogel was used in the treatment of a rat sciatic nerve defect, a significant amount of cytoskeletal actin organized alongside the peptide was detected, with an enhancement of motor/sensory function and optimal axonal regeneration. More recently, Nam and co-workers synthesized a hybrid hydrogel containing the self-assembled *β*-peptide betaVhex (hKhKhVhKhE-hVhFhFhVhK-hEhVhFhFhV-hKhEhVhYhK) and carbon nanotubes (CNTs), in order to be able to interact with neurons [164]. CNTs were used to promote neural signal transmission. The composite showed good biocompatibility and its mechanical properties fitted well with those of the native tissue, resulting in the complete integration of the composite. A dramatic neural signal enhancement was detected during seizures in the epidural tissue. When this scaffold was administered to the cortex layer of epileptic mice through injection, a 2.4-fold signal amplification was observed.

### 3.3. Cartilage Regeneration

**Figure 4:**
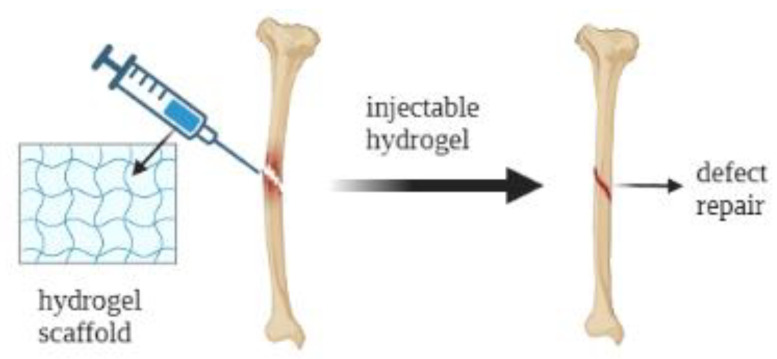
Schematic illustration of application of hydrogels for bone defect repair.

Aging and some diseases such as trauma and joints degeneration could cause lesions in chondral and osteochondral tissues, which is related to the disappearance of vascular, neural, and lymphatic frameworks as well as progenitor cells. Therefore, repairing articular cartilage is extremely challenging. These conditions even cause further decline of the articular cartilage and may lead to disability [165,166]. Using self-assembling peptides could represent a valuable strategy for dealing with the difficulties of cartilage regeneration (see Figure 4) [167]. In 2022, Ye et al. [168] constructed a LIANAK peptide (CM) mimicking the transforming growth factor β (TGF-β) and they connected this sequence to the well-known self-assembling RADA16 peptide. The grafted peptide (RADA16-CM) was able to stabilize TGF-β, which induces the differentiation of mesenchymal stem cells as well as the sprinkle of collagen II. The authors showed that the fabricated RADA16-CM hydrogel enhances the expression of chondrogenic genes and ECM formation. The constructed hydrogel was then paired with decellularized cartilage ECM for the preparation of a scaffold for the repair of articular cartilage. The fabricated composite showed adequate stability and bioactivity. In addition, its ability to induce cartilage tissue regeneration was very promising. In this research, Ye and co-workers [168] showed that by the incorporation of unstable TGF-β1 within the CM peptide sequence, the final stable product could be used for in situ cartilage regeneration. In 2021, Zanotto et al. described a KLD hydrogel linked to the trypsin treatment growth factor as an alternative for microfracture reinforcement method, which is a high-cost technique for current cartilage repair [169]. The results revealed that trypsin treatment in combination with the hydrogel was able to improve microfracture augmentation. In small animal models this strategy overall improved cartilage regeneration. In addition, a moderately improved joint effusion and subchondral bone sclerosis were observed. From a microscopic aspect, this treatment was able to improve various histologic variables and the quality of the repaired tissue was overall improved. This research showed that this therapeutic strategy for microfracture augmentation is a cost-effective way to improve cartilage healing, especially in patients that are more active. Recently, Thomas et al. fabricated a peptide hydrogel consisting of an amyloid-inspired amphiphile which self-assembled into nanofibers and was inserted in a polysaccharide network of carboxymethyl cellulose dialdehyde and carboxymethyl chitosan by a Schiff base synthesis [170]. It is worthwhile to mention that non-covalent interactions in hydrogel structures play essential roles in the modulations of their mechanical properties, necessary for designing cartilage scaffolds. The ability of the fabricated scaffolds to promote chondrogenesis was evaluated in vitro using human chondrocytes. Results revealed the improvement of cell growth and production of cartilage-specific ECM, showing the ability of the construct to aid cartilage tissue regeneration and confirming the importance of recreating a suitable microenvironment for optimal results. In 2022, Wang and co-workers reviewed the use of self-assembling peptide hydrogels, including KLD-12, RADA16 and IEIK13, as suitable candidates for the regeneration of cartilage [171]. Such hydrogels could have a significant clinical role in the future by providing the conditions for cell morphology and viability maintenance, increasing the release of cartilage-specific ECM, and repairing defects *in situ*. Although they may have some limitations, the functionalization of their structure is a strategy to promote desirable properties. For example, by the introduction of short functional peptides, they would be able to show more powerful therapeutic effects. In addition, cells and cytokines take part in the repair activities after cartilage damage. Composite hydrogels containing cells or cytokines can have improved therapeutic functions, enhancing the proliferation and chondrogenic differentiation of the surrounding cells. In another recent research, Huang and co-workers [172] used a PFSSTKT sequence with an affinity to BMSCs and modified with chondrocyte ECM. The above structure was combined with a Gelatin methacrylate hydrogel for evaluating the ability of the molded scaffolds to repair cartilage defects. The results of the in vitro experimentation evidenced that the porosity and pore-size of the scaffold were suitable, and this composite provided a 3D microenvironment which was able to promote cell adhesion, growth and chondrogenic differentiation. In addition, the results supported the conclusion that the composite hydrogel may adjust the migration of BMSCs. In vivo experiments were conducted in rabbits and demonstrated that the composite scaffold was able to induce the recruitment of endogenous mesenchymal stem cells to the defect site after two weeks. Therefore, it seems that the strategy of combining endogenous cell recruitment and chondrogenesis could be applicable for repairing irregular cartilage defects. In 2021, Dufour et al. [173] conducted a pilot study on the fabrication of an IEIK13 peptide hydrogel in combination with articular chondrocytes supplied with a chondrogenic cocktail consisting of BMP-2, insulin, and triiodothyronine, to investigate its ability in restoring large cartilage defects in the femoral condyles of cynomolgus monkeys. In vitro results confirmed that the synthesized IEIK13 composite hydrogel was able to induce the production of a sufficient amount of cartilage articular chondrocytes treated with triiodothyronine. A contrast-enhanced micro-computed tomography technique, histological analysis and immunohistochemical staining of the condyles were employed to monitor implant integration *in vivo*. Based on the results, IEIK13 implants were suitable for full-thickness treatment of injured cartilage, loaded or devoid of chondrocytes. Another peptide hydrogel scaffold capable of chondrocyte encapsulation, named KLD-12, was fabricated by Kisiday et al. [174]. This hydrogel was able to support the chondrocyte phenotype and enhance the production of cartilage-like ECM. Furthermore, the accumulation of glycosaminoglycan (GAG) as a function of time, along with a stiffness increase provided additional evidence for the formation of healthy cartilage. In addition, the authors investigated the chondrogenesis of MSCs on this type of scaffold. In another research conducted by Li et al. [175], a HAVDI-modified KLD-12 hydrogel was studied. HAVDI is a bioactive peptide sequence, which promotes cell adhesion and chondrogenesis. The hydrogel showed significant biocompatibility with MSCs (viability: 94%). The results showed that between a KLD-12 self-assembled hydrogel and the one incorporated with HAVDI, the second one was more successful at inducing an increased expression of chondrogenic genes (collagen II, aggrecan, sox9) and it led to chondrogenic differentiation within 14 days. Regarding the involved molecular mechanism, it was found that the KLD-12 hydrogel/HAVDI composite hindered the β-catenin localization in the nucleus on day three and it was suggested that the inhibition of normal Wnt/β-catenin signaling is the reason of chondrogenesis enhancement. Florine et al. designed RADA16 hydrogel scaffolds incorporating the fusion protein Heparin-binding insulin-like growth factor 1 (HB-IGF-1) and they showed that this scaffold promotes the production of sulfated GAG and hydroxyl proline within hydrogels seeded with chondrocytes [176]. In recent research, Liebesny and co-workers prepared a KLD-12 peptide hydrogel functionalized with HB-IGI-1. Then they encapsulated chondrocytes within the fabricated scaffold and loaded the hydrogel with trypsin [177]. The function of the enzyme was removing the sulfated GAG from the edge of defect sites, thus allowing the chondrocytes encapsulated in the scaffold to migrate towards adjacent cartilage annuli. They reported that the use of this composite scaffold leads to a significant enhancement of proteoglycan production degree, GAG deposition within the chondrocyte-seeded scaffold and integration with native cartilage tissue in four weeks. The functionalization of the RADA16 peptide hydrogel with the functional motif PFS (PFSSTKT), which is a bone marrow homing peptide providing conditions for stem cells binding [178,179], and encapsulation of the cellular cartilage matrix within the hydrogel scaffold was described to investigate the recruitment of endogenous stem cells for cartilage regeneration [180]. Results revealed that the RADA16/PFS peptide scaffold supports the attachment and MSCs’ chondrogenic differentiation in vitro. In addition, it was shown that the expression level of chondrogenic genes (aggrecan, Sox9, and Col2) of MSCs seeded in the scaffold.

At present, cartilage tissue engineering is developing rapidly. Hydrogels are widely used in tissue engineering because of their similarity to ECM. They possess excellent biological characteristics, an injectable ability for cartilage in situ repairing and play a therapeutic role.

### 3.4. Bone Regeneration

Autogenous bone is considered as the “gold standard” in bone tissue engineering practices. However, although several kinds of scaffolds used for the reconstruction of bone tissue, such as ceramics and metal sand alloys, are available, the application of autografts is limited by availability and donor site morbidity. The design of artificial scaffolds with the required osteoinductive or osteogenic properties is a challenging process [181,182]. Recently, an innovative injectable self-healing hydrogel system for enhancing vascularization during the regeneration of irregular bone defects was constructed [183]. The results revealed that the fabricated GMO hydrogel showed an optimal injectability and is suitable for fitting uneven defects, thanks to the presence of dynamic imine bonds between gelatin methacryloyl and oxidized dextran. The hydrogel bioactive properties were tailored through the incorporation of KP (BMP2 knuckle epitope derived peptide) and KLT peptides, which possess osteogenic and angiogenic properties; these were released at appropriate rates. In vitro results revealed that the composite hydrogel improved the osteogenic differentiation of BMSCs and angiogenetic properties of HUVECs significantly. According to the in vivo results, the above-mentioned peptides synergistically cooperated in stimulating ossification in rat calvaria. It was concluded that the use of such a peptide-loaded hydrogel could be considered as an effective strategy for bone tissue engineering with minimal invasiveness. Stüdle and co-workers recently studied bi-layer PEG hydrogels [184]. One layer was embedded with endochondral ossification cells like BMSCs and TGF-β or BMP-2 growth factors. In another layer of the gel, chondrogenesis cells like nasal chondrocytes (NCs) were encapsulated and this composite was implanted in mice without pre-culturing. The goal of the research was to ascertain if these two cell types embedded in a bi-layered hydrogel could directly lead to the formation of osteochondral tissues *in vivo*. Results revealed that the layers containing BMSCs produced ossicles containing bone marrow. In addition, the NC-embedded layers generated cartilage tissue, whose phenotype was lasting when BMP-2 was present. This research revealed that the orderly connected osteochondral composites have a high potential to be used as a model for the development of cartilage bone interface. The fabrication of a supramolecular bioactive material consisting of an amphiphilic peptide along with the IKVAV motif to lead neural transdifferentiation of BMSCs was reported by the Ji group [185]. The synthesized peptide was able to self-assemble and form supramolecular nanofibers that enforce the commitment of neuroectodermal lineage after 1 week. This was confirmed by the upregulation of the neural progenitor gene Nestin and glial fibrillary acidic protein. However, results demonstrated that a significantly higher expression of different neuronal markers was observed after two weeks. BMSCs’ growth within the fabricated composite lead to a polarized cytoskeletal architecture decreasing the cellular size, which is similar to neuron cells. This research could pave the way for a transdifferentiation of adult human BMSCs into neuronal lineage. In 2019 Panek et al. [186] fabricated a (RADA16) hydrogel impregnated with different amounts of dexamethasone (DXM) (4 × 10^−3^–10^−5^ M). In order to test the fabricated composite, MSCs were isolated and cultured to be loaded on the composite scaffold in a perfusion bioreactor. Scanning electron microscopy and histology were employed to analyze tissues, examining the morphologies of cells, ECM, and minerals. Real-time polymerase chain reaction and immunocytochemistry were employed to quantify the markers of osteogenic differentiation. Osteoblast-related markers were quantified to confirm osteoblast differentiation. The results of the immunocytochemical analysis of collagen I supported the conclusion that the optimal concentration of loaded DXM is 4 × 10^−4^ M, which provides the conditions for the production of the best-engineered bone tissue for 21 days at a perfusion rate of 0.1 mL/min. Misawa et al. showed that a commercial RADA16 hydrogel scaffold (PuraMatrix^TM^) aided the formation of new bone in an animal calvaria defect model [187]. It was also found by He et al. that both D-RADA16 and L-RADA16 hydrogel scaffolds could promote bone regeneration in a femoral condyle defect model with no functionalization [188]. This research group also designed a D-RADA16 peptide hydrogel modified with the cell adhesion motif RGD along with the basic fibroblast growth factor (bFGF) [189]. It was shown that the fabricated composite could promote osteoblasts’ growth and outspread, inducing neovascularization in vivo [190,191]. Results demonstrated that after treatment of femoral condyles with modified peptide hydrogels, a significant decrease in defect domains during the eight weeks was observable, even in the absence of bFGF. In addition, a whole section containing numerous bone defects was entirely repaired within 12 weeks.

The mentioned approaches employed long peptidic sequences as well as other motifs or functionalizations for the fabrication of hydrogel scaffolds; however, there are some reports using short peptide sequences. For example, a biodegradable hydrogel comprising a self-assembling FEFEFKFK octapeptide with no functional motifs was described [192]. The hydrogel was compatible with MSCs and it supplied them with a favorable microenvironment. The significant production of common osteogenic markers along with a paramount deposition of hydroxyapatite following the treatment with osteogenic medium confirmed the osteogenic differentiation of human MSCs after 12 days, which suggested successful bone formation. It was also concluded that due to its uncomplicated sequence, affordability and positive in vivo conduct, the use of such an octapeptide could be considered as a good strategy for bone engineering and even clinical use [3]. In another investigation, the dipeptide fluorenyl methoxycarbonyl diphenylalanine (FmocFF) along with the alginate was reported to afford hydrogel formation and bone regeneration induction [178,179]. The fabricated hydrogel was biocompatible and it induced the osteogenic differentiation of MC3T3-E1 preosteoblast cells. Moreover, the composite was responsible for the rise in the expression level of several osteogenic genes and calcification [193]. Another hydrogel, consisting of an alginate modified with the peptides KLT and RGD was able to contemporarily induce both angiogenesis and osteogenesis [194]. The effects of the grafted peptides were calcification and proangiogenesis. The composite was tested in a rat calvarial defect model, leading to higher angiogenesis and ossification than the control.

The reported results in this field demonstrated that an injectable peptide-based hydrogel is able to fulfill the multifold requirements of applications preserving a good biocompatibility and appropriate drug properties, and that it possesses a significant potential for bone tissue engineering application.

## 4. Conclusions

In this review paper we have summarized a wide range of peptide hydrogels that are frequently used to-date, or will potentially be useful in tissue engineering by focusing in particular on mechanical properties, as well as on biodegradability and bioactivity. These biomaterials resembled native cellular composition and morphology; they can exhibit several biofunctional features and provide favorable micro-environments for cell adhesion, proliferation, migration, and differentiation. A critical element in virtually all tissue engineering approaches is the chemical feature of the polymer scaffold which potentially mimics many roles of extracellular matrixes found in tissues. In recent years, various peptide sequences have been fabricated consisting of different types of natural amino acids used in hydrogel structures with inherent biocompatibility. However, some of their properties must be tailored by improving conditions such as self-assembly behavior, mechanical properties, and bioactivity. In addition, for tissue engineering applications, different peptide sequences have been studied such as KLD-12, RADA16, and HAVDI because of their significant properties, which make them appropriate for designing hydrogel scaffolds. Recently, most peptide hydrogels that are used for tissue engineering are functionalized with different peptide sequences, drugs, growth factors or motifs to increase their efficiency. In addition, advances made regarding stem cells has promoted the possibility for them to be more available than before for tissue regeneration applications. This condition enables the use of autologous cell sources. It also should be mentioned that a deep understanding of the interplay between peptide scaffolds and stem cells and their outcomes on cell differentiation are providing worthwhile information for the future development of more specific peptidic scaffolds with significant features. Apart from the advances in designing peptide hydrogels for tissue regeneration described in this review, there are still some challenges especially for the use of hydrogels in the clinical environment. For example, a lack of information regarding the outcomes of mechanosignaling on the destination of different stem cells hinders the design of appropriate viscoelastic characteristics. Additionally, various peptide scaffolds with numerous functional components have been fabricated to enhance the effects of tissue regeneration but some drawbacks such as their toxicity on cells and tissues and possible negative reactions on implantation sites prevents their use in biological media. As a plan for the future, technical challenges of peptide hydrogels along with their scientific issues should be revised by considering: (1) the importance of functional and structural batch to batch reproducibility of peptide hydrogels, especially for those containing cells and large biomolecules; (2) a fast and large-scale fabrication of complex cell-free peptide hydrogels while maintaining reproducibility and functionality; since mass production of cell-encapsulated hydrogel scaffolds is arduous, in the case they could be developed for personalized medicine; (3) the sterilization process of peptide hydrogels for medical use while avoiding disruption in assembly and functionality. Overall, despite all the mentioned challenges in the field of peptide hydrogel scaffolds, continuous progress will finally pave the way for fruitful clinical applications of tissue regeneration products.

In conclusion, we believe no one material will be able to satisfy all design parameters in all applications, but a wide range of materials will find uses in various tissue engineering applications.

## Figures and Tables

**Table 1:** Some of the most common bioactive motifs used in the structure of peptide hydrogels for tissue engineering applications.

Peptide Motif	Bioactivities	Origin	Integrin(s), Cell/Proteins Binded	Ref.
RGD	Cell adhesion enhancement	ECM proteins (Fibronectin, collagen, vitronectin)	α5β1, α8β1, αvβ1, αvβ3, αvβ3, αvβ5, αvβ6, αvβ8, αIIbβ3	[105,106]
IKVAV	Cell growth enhancing along with neural differentiation promoting and nerve regeneration	Laminin (α1 chain)	α3β1, α4β1, α6β1	[107,108]
YIGSR	Enhancement of cell adhesion and migration	Laminin (β_1_ chain)	α3β1, α4β1, α6β1	[109]
PHSRN	Cell adhesion enhancement	ECM proteins (same as RGD)	α5β1	[110,111]
KLPGWSG	Neuronal differentiation enhancement	Stem cells proteins	Neural stem cells (NSCs)	[112]
PFSSTKT	Neural cell proliferation and differentiation; human adipose stem cell homing promotion	Bone marrow homing	Nerve and spinal cord	[113]
KPSS	Promotion of accumulation of ECM; induction of bone marrow MSCs migration	Morphogenic proteins derived from bone	β-Kdo-transferases	[114,115]
Substance P (RPKPQQFFGL)	Cartilage regeneration improvement; wound healing promotion	Neuropeptides (endogenous type)	β2	[116]
Link N (DHLSDNYTLDHDRAIH)	Stabilization of proteoglycan aggregates	Derived from link protein exists in disk tissues	N/A	[117]
REDV	Induction of angiogenesis; enhancement of endothelial cell adhesion	Fibronectin	α4β1	[118]
KLT	Acts as an analog of VEGF.	VEGF mimetic peptide	VEGF receptors	[103]
PRG	Possesses homology to the lipid phosphate phosphatases (LPPs) in nervous system	Integral membrane protein	β1 (Protein phosphatase 2A, PP2A)	[119]
SNVI	Displaying bone morphogenic peptide-7 (BMP-7) bioactivity	Bioactive sequence of BMP-7	N/A	[120]
SVVYGLR	Angiogenesis, production of collagen III, and fibroblast differentiation into myofibroblasts	Osteopontin protein	α4β1, α9β1, α4β7	[121,122]
HAVDI	Cell adhesion	N-Cadherin (calcium-dependent cell-cell adhesion) protein	Extracellular domain 1 (ECD1) of N-cadherin protein	[123]
QLK	Covalent binding to transglutaminase to protect GFs from proteolytic	N/A	N/A	[124]
LRK	Joining angiogenic inducers (HGF, and VEGF)	N/A	Kinases in plants	[124]

**Table 2:** Main features of common self-assembling peptides and examples of their application.

Self-Assembling Peptides	Abbreviation	Self-Assembly Mechanism	Higher-Order Structure	Application and Features
CH_3_CO-RATARAEARATARAEA-CONH_2_	RATEA16	Hydrophobic interactions, intermolecular hydrogen bonds, electrostatic interactions	β-sheet nanofibers	Use in controlled release of therapeutics through pH-response and in diffusion release [129]
CH_3_CO-(RADA)_4_-CONH_2_	RADA16	Hydrophobic interactions, intermolecular hydrogen bonds, electrostatic interactions	Antiparallel β-sheet structure	Stable fibril units with high water content for making three-dimensional scaffolds for cell culture [130]
Fmoc-DIKVAV	-	π-π, and electrostatic interactions, hydrogen bonds	β-sheet structure	Petide-based biomaterial combined with polysaccharides to afford a wide range of achievable physico-chemical properties [131]
CH_3_CO-KLDLKLDLKLDL-CONH_2_	KLD-12	Electrostatic interactions, hydrogen bonds	β-sheet structure	Protein-based nanostructured templates with enhanced versatility for tissue engineering of bones and teeth [132]
CH_3_CO-IEIKIEIKIEIKI-CONH_2_	IEIK-13	Hydrophobic and electrostatic interactions, hydrogen bonds	β-sheet structure	Hemostatic potential and safety of RADA16 and IEIK13 for hemostasis in the rat brain [133]
FEFEFKFK	-	π-π, and electrostatic interactions, hydrogen bonds	β-sheet structure	The self-assembly and gelation properties of FEFEFKFK depend on pH media [134]

## Data Availability

Not applicable.
